# Sepsis Diagnosis in the Intensive Care Unit: A Comparative Study of Rapid Molecular Diagnostics and Conventional Blood Cultures

**DOI:** 10.3390/biomedicines14020479

**Published:** 2026-02-22

**Authors:** Dragana Unic-Stojanovic, Nikolina Kangrga, Ivana Cirkovic, Irina Malesevic, Ivana Djokovic Mrdakovic, Jovan Petrovic, Milovan Bojic

**Affiliations:** 1 Faculty of Medicine, University of Belgrade, 11000 Belgrade, Serbia; 2 Institute for Cardiovascular Diseases Dedinje, 11000 Belgrade, Serbia; nikolinakangrga@gmail.com (N.K.);; 3 Institute of Microbiology and Immunology, University of Belgrade, 11000 Belgrade, Serbia

**Keywords:** sepsis diagnosis, T2Bacteria, T2Resistance, blood culture, targeted antimicrobial therapy

## Abstract

**Background:** Sepsis remains a leading cause of morbidity and mortality worldwide, where timely and accurate pathogen detection is critical for improved outcomes. Conventional blood cultures are the gold standard but are limited by prolonged turnaround times and suboptimal sensitivity, often delaying targeted therapy. **Methods:** This single-center retrospective study evaluated the diagnostic performance and clinical utility of the T2Bacteria and T2Resistance Panels compared with conventional blood cultures in 30 adult patients admitted to the cardiovascular intensive care unit with a suspected bloodstream infection. **Results:** The T2Bacteria Panel demonstrated high diagnostic accuracy for on-panel organisms (100%), detecting all cases of *Klebsiella pneumoniae*, *Acinetobacter baumannii*, and *Pseudomonas aeruginosa,* while blood cultures detected 9 of 12 on-panel infections. In contrast, two off-panel organisms were isolated from five patients exclusively by blood cultures, highlighting the complementary roles of both methods. Importantly, antimicrobial therapy was modified in 6 of 10 T2-positive patients (60%) based on T2 results, preceding blood culture reporting by a median of more than 100 h. **Conclusions:** These findings underscore the value of T2 assays in enabling earlier, evidence-based therapeutic decisions and supporting antimicrobial stewardship. While limited by the sample size and single-center design, these findings—consistent with pathogen distributions reported in European ICU settings—suggest that integrating T2-based diagnostics into cardiovascular ICU workflows may enhance early therapeutic decision-making and antimicrobial stewardship.

## 1. Introduction

Sepsis is one of the most pressing global health challenges, with an estimated 49 million cases and 11 million deaths worldwide annually, accounting for nearly 20% of all deaths [1]. Defined as life-threatening organ dysfunction caused by a dysregulated host response to infection, sepsis can rapidly progress to septic shock, a state in which mortality rates reach 40–80% [2]. Despite major advances in supportive care and antimicrobial therapy, the early identification of bloodstream infections (BSIs) and timely initiation of effective treatment remain critical determinants of patient survival. It has been well documented that each hour of delay in the administration of appropriate antimicrobials after the onset of septic shock decreases survival by approximately 7.6% [3]. Consequently, the need for rapid and accurate pathogen identification methods is paramount.

Blood culture (BC) has long been considered the gold standard for the diagnosis of bacteremia and fungemia. However, this conventional method is time-consuming, as the growth of microorganisms in culture requires hours to days depending on the pathogen, often leading to a turnaround time of three to five days before species identification and susceptibility results are available [4]. Moreover, the sensitivity of BC is suboptimal, with positive results obtained in only about 50% of clinically suspected BSIs [5]. This sensitivity is further compromised by prior antimicrobial therapy, fastidious or non-cultivable organisms, and low-level bacteremia [6]. Additionally, blood culture is susceptible to contamination, which may lead to false-positive results, unnecessary antimicrobial exposure, and prolonged hospitalization [7]. These shortcomings highlight the urgent need for novel diagnostic technologies enabling the faster, more sensitive, and more accurate detection of bloodstream pathogens.

Various diagnostic techniques, such as PCR-based assays, multiplex real-time PCR platforms, electrospray ionization mass spectrometry (PCR/ESI-MS), and metagenomic sequencing, are aimed at overcoming the limitations of BC [4,7]. These technologies are designed to enable the earlier detection of pathogens directly from whole blood, circumventing the culture step and significantly reducing the time to results. While promising, many of these early platforms are limited by variable sensitivity, high costs, and workflow complexity [8].

Among the most notable innovations in this field is the T2 magnetic resonance (T2MR) technology developed by T2 Biosystems—the first and only commercially available culture-independent diagnostic assay capable of detecting common bacterial pathogens directly from whole blood [9]. The T2Bacteria Panel detects a subset of clinically important pathogens, including *Klebsiella pneumoniae*, *Acinetobacter baumannii*, and *Pseudomonas aeruginosa*, which represent key Gram-negative members of the ESKAPE group and are commonly associated with multidrug-resistant bloodstream infections in critically ill patients [10]. These organisms account for more than 40% of BSIs worldwide and are associated with longer hospital stays, higher treatment costs, and increased mortality compared to non-ESKAPE pathogens [11].

In addition to bacterial detection, the T2 platform has expanded to include the T2Resistance Panel, capable of identifying key resistance genes directly from blood within hours [12]. Preliminary studies suggest that pairing T2Bacteria with T2Resistance could provide simultaneous information on both pathogen identity and resistance determinants, enabling truly personalized and timely antimicrobial therapy [13].

The clinical utility of the T2Bacteria Panel has been supported by multiple prospective, multicenter studies [14,15]. Early and reliable pathogen detection can facilitate a more rapid transition from empirical broad-spectrum therapy to targeted antimicrobial regimens, thereby reducing unnecessary antibiotic exposure and its attendant risks, including the emergence of resistance, drug toxicity, and disruption of the microbiome [16]. Nonetheless, the heterogeneity of study designs, patient populations, and endpoints has led to some conflicting results, particularly regarding mortality [13]. This underscores the need for further rigorous clinical trials and real-world evaluations to establish the definitive role of T2Bacteria in routine practice.

Despite growing evidence supporting rapid molecular diagnostics in general intensive care unit and emergency department populations, data specifically addressing cardiovascular intensive care units remain limited [17,18]. Patients undergoing cardiac surgery represent a distinct clinical population characterized by pronounced sterile inflammatory responses, frequent prior antibiotic exposure, and a high prevalence of multidrug-resistant Gram-negative pathogens, all of which may influence diagnostic performance and the interpretation of microbiological results.

Furthermore, few real-world studies have evaluated the antimicrobial stewardship impact of T2-guided diagnostics, particularly regarding early therapy modification and de-escalation strategies in post-cardiac surgery patients. By focusing on a cardiovascular ICU cohort with high baseline antimicrobial exposure, the present study addresses these setting-specific gaps and provides clinically relevant data on diagnostic accuracy, resistance detection, and therapeutic decision-making in this underrepresented population [17,18].

The aim of the present study was to evaluate the diagnostic accuracy of the T2Bacteria Panel in patients with suspected bloodstream infections in a cardiovascular intensive care unit and to assess its clinical impacts on antimicrobial therapy modification, resistance gene detection, and the time to targeted antimicrobial treatment compared with conventional blood cultures.

## 2. Materials and Methods

This retrospective observational study was conducted at the Department of Anesthesiology and Intensive Care, Institute for Cardiovascular Diseases Dedinje (Belgrade, Serbia). The study included patients admitted to the intensive care unit (ICU) between June 2024 and June 2025. At the time of diagnostic sampling, all patients were receiving empirical broad-spectrum antimicrobial therapy in accordance with institutional sepsis management protocols. A total of 30 patients were enrolled, all of whom underwent simultaneous diagnostic testing with the T2Bacteria Panel and conventional blood cultures as part of their standard clinical management [1,2]. The study was approved by the Institutional Review Board (Ethics Committee) of the Institute for Cardiovascular Disease Dedinje (N. 5372/2025, approval date: 3 October 2025).

The study cohort comprised adult patients admitted to the ICU either on an elective or emergency basis following cardiovascular surgery. Demographic characteristics were systematically collected, including sex, age, type of admission, type of surgical procedure, and comorbidities.

For each patient, routine biochemical parameters were retrieved at three critical time points: at admission to the hospital, at the time of T2Bacteria sampling, and at the time when conventional blood culture results became available. Biochemical parameters that were collected were C-reactive protein (CRP), procalcitonin, leukocyte count, lactate concentration, and renal function parameters, while the collected clinical parameters were body temperature, hemodynamic status at the time of suspected infection, heart rate, and mean arterial pressure. Clinical severity at the time of suspected infection was assessed using the Glasgow Coma Scale (GCS) and Sequential Organ Failure Assessment (SOFA) score. SOFA scores were calculated using standard criteria (MDCalc^®^, https://www.mdcalc.com, accessed on 4 October 2025), including respiratory function (PaO_2_/FiO_2_ ratio), coagulation (platelet count), liver function (bilirubin), cardiovascular status, neurological status (GCS), and renal function (creatinine level). In addition, peak values of inflammatory markers (CRP, procalcitonin, leukocyte count) were documented.

Whole-blood samples for the T2Bacteria Panel (T2 Biosystems) were collected upon clinical suspicion of bloodstream infection. The exact time of sampling and the interval until result reporting were recorded. For each test, the presence or absence of targeted bacterial pathogens was documented, and positive findings were cross-validated against those of conventional microbiological cultures when available. The T2Resistance Panel was performed in all patients in whom a pathogen was isolated from the T2Bacteria Panel, providing direct-from-blood detection of major antimicrobial resistance genes, including carbapenemases (*blaKPC*, *blaNDM*, *blaVIM*, *blaIMP*, *blaOXA-48* group), extended-spectrum β-lactamases (*blaCTX-M-14/15*), AmpC (*blaCMY/DHA*), vancomycin resistance genes (*vanA/B*), and methicillin resistance genes (*mecA/C*). Results were analyzed in relation to both T2Bacteria and conventional culture findings.

Conventional blood cultures were obtained simultaneously or within a narrow time frame after T2 sampling, following standard aseptic procedures. Blood culture samples were processed at the Laboratory for Bacteriology, Institute of Microbiology and Immunology, Faculty of Medicine, University of Belgrade, following the laboratory’s standard operating procedure for blood culture diagnostics. Aerobic and anaerobic bottles were incubated in the BacT/ALERT^®^ blood culture system (bioMérieux, Marcy-l’Étoile, France). When a bottle signaled a positive result, a Gram stain was performed, and the sample was subcultured onto blood agar and MacConkey agar plates. For microbial identification, MALDI-TOF MS (Bruker Biotyper, Bruker Daltonics, Bremen, Germany) was applied using a rapid protocol based on 4 h microcolony growth, rather than overnight cultures, as previously described [19]. Depending on organism identification, antimicrobial susceptibility testing was carried out using the VITEK^®^ 2 system (bioMérieux), applying cards appropriate for the detected species. For *Enterobacterales*, the AST-N439 card was used; for *Pseudomonas* spp., *Acinetobacter* spp., and other non-fermentative Gram-negative bacilli, the AST-N440 card was used; and for Gram-positive cocci (*Staphylococcus* spp. and *Enterococcus* spp.), the AST-P592 card was used. Colistin susceptibility was determined using broth microdilution with the commercial MIC Test Strip Broth Microdilution kit (Liofilchem^®^, Roseto degli Abruzzi, Italy). All results were interpreted according to the EUCAST Clinical Breakpoints (www.eucast.org, accessed on 11 October 2025). Data recorded included the time of sampling, the interval until results were reported, and the identification of microbial isolates. Concordance between T2Bacteria results and blood culture findings was assessed for each patient.

The duration of empirical antimicrobial therapy was recorded for all patients, along with subsequent treatment modifications. Specific attention was given to whether adjustments in antimicrobial therapy were made after the receipt of T2Bacteria and/or T2Resistance results and whether these changes preceded the receipt of conventional blood culture results. Blood culture was used as the primary reference standard for the calculation of diagnostic performance parameters. In cases with discordant results between T2Bacteria and blood culture (T2+/BC− or T2−/BC+), clinical adjudication was performed based on the overall clinical course, response to antimicrobial therapy, microbiological findings from other body sites, imaging results, and repeat culture data when available. For statistical calculations, discordant T2-positive/blood culture-negative cases were categorized according to the blood culture results as the reference standard; however, their clinical relevance was interpreted separately and discussed in detail.

Cases of incomplete data were defined as missing key clinical information, microbiological results, or treatment data necessary for outcome and performance analysis. Cases with incomplete datasets were excluded from the final analysis.

The primary outcome of interest was the diagnostic performance of the T2Bacteria Panel compared with conventional blood cultures, expressed in terms of sensitivity, specificity, positive predictive value (PPV), and negative predictive value (NPV). Secondary outcomes included the following: (I) time to pathogen identification for T2 versus conventional cultures; (II) concordance between T2 and culture results; (III) detection of resistance genes by the T2Resistance Panel; and (IV) clinical impact reflected in the duration and modification of empirical therapy.

All collected variables were entered into a structured database. Categorical variables (e.g., pathogen detection, concordance rates, resistance gene identification) were expressed as absolute numbers and percentages. Continuous variables (e.g., time to result, inflammatory marker levels, duration of empirical therapy) were summarized using medians and interquartile ranges (IQRs) due to non-normal distributions. The diagnostic performance of the T2Bacteria Panel was evaluated against blood culture results as the reference standard, calculating the sensitivity, specificity, PPV, and NPV. Comparisons of the time to results between T2 and conventional cultures were performed using non-parametric statistical tests (Mann–Whitney U or Wilcoxon signed-rank test, as appropriate). A *p*-value < 0.05 was considered statistically significant. All statistical analyses were performed using IBM SPSS Statistics for Windows, version 29.0.1.0. [20]. Given the limited sample size, this study was designed as an exploratory analysis. The study was not powered to detect differences in hard clinical outcomes such as mortality; therefore, all outcome analyses should be interpreted descriptively and as hypothesis-generating in nature.

## 3. Results

### 3.1. Patient Demographics and Baseline Characteristics

A total of 30 patients were included in the study cohort. The main characteristics of the study population are summarized in Table 1. The median age was 65.5 years, with a predominance of male patients (*n* = 19, 63.3%) compared to females (*n* = 11, 36.7%). Six patients (20.0%) were admitted on an emergency basis, while the remaining 24 patients (80.0%) were elective admissions. Nearly all patients (*n* = 29, 96.7%) underwent major cardiovascular surgical procedures, whereas one patient (3.3%) was admitted for vascular surgery.

All T2Bacteria and blood culture samples yielded valid results. No test failures, invalid assays, or contaminated blood culture samples were observed. Therefore, all collected samples were included in the final performance analysis.

At the time of T2Bacteria Panel sampling, patients displayed clinical and laboratory evidence of a systemic inflammatory response. The mean body temperature across the cohort was 38.1 °C, with most patients presenting with febrile episodes. Inflammatory biomarkers were elevated, with a median leukocyte count of 12.45 × 10^9^/L and a median CRP concentration of 142 mg/L. These findings reflected the severity of the underlying systemic response and provided the clinical rationale for microbiological investigation.

All patients included in the study were receiving empirical broad-spectrum antimicrobial therapy at the time of diagnostic sampling. This reflects routine clinical practice in critically ill post-cardiac surgery patients with suspected sepsis. Despite universal prior antibiotic exposure, the T2Bacteria Panel demonstrated preserved diagnostic yields for on-panel pathogens, whereas the blood culture’s sensitivity was reduced, with several bloodstream infections not detected by conventional culture methods.

The levels of inflammatory biomarkers were compared between diagnostic groups. Patients with positive T2Bacteria results demonstrated higher median procalcitonin and CRP values compared with T2-negative patients; however, due to the limited sample size, these differences did not reach statistical significance. Similarly, biomarker levels were descriptively higher in blood culture-positive patients compared with blood culture-negative patients. Although elevated biomarker levels were associated with microbiologically confirmed infections, they were not independently predictive of T2 positivity.

### 3.2. Microbiological Results

The T2Bacteria Panel was positive in 10/30 patients (33.3%), while blood cultures were positive in 12/30 patients (40%). Concordance between the two diagnostic methods was achieved in 24/30 cases (80.0%). Specifically, eight patients (26.7%) were concordantly positive (T2+/BC+), and sixteen (53.3%) were concordantly negative (T2-/BC-). Discordant results occurred in six patients (20.0%): in two cases (6.6%), the T2Bacteria Panel was positive while blood culture was negative (T2+/BC-), whereas, in four cases (13.3%), blood culture was positive and T2 negative (T2-/BC+) (Table 2).

Sixteen patients (53.3%) had concordantly negative results (T2-/BC-). In this group, clinical assessment frequently identified alternative causes of the systemic inflammatory response, including non-infectious postoperative inflammation, infections originating from non-bloodstream sites, or transient inflammatory syndromes. In a subset of these patients, antimicrobial therapy was subsequently de-escalated or discontinued following negative microbiological findings and clinical stabilization. These observations support the potential value of combined negative T2 and blood culture results in guiding diagnostic reassessment and optimizing antimicrobial stewardship.

Both patients with T2+/BC- were receiving empirical broad-spectrum antimicrobial therapy at the time of sampling. T2Bacteria detected *K. pneumoniae* in one case and *P. aeruginosa* in the other. In both patients, antimicrobial therapy was modified shortly after T2 result availability and was followed by a clinical improvement, including a reduction in inflammatory markers and hemodynamic stabilization. Blood cultures remained negative, and no additional bloodstream pathogens were isolated on repeat cultures. In one patient, the same pathogen detected by T2 was subsequently identified from a respiratory tract sample, supporting the clinical relevance of the T2 finding. Both cases were clinically confirmed as true bloodstream infections based on the clinical presentation and favorable response to targeted therapy.

In patients with T2-/BC+ results, all identified organisms were off-panel pathogens, including *B. cepacia* and *S. epidermidi*s. Blood cultures became positive after prolonged incubation. These patients presented with moderate to severe clinical illness, reflected by elevated SOFA scores and inflammatory markers at the time of sampling. Antimicrobial therapy in these cases was guided by blood culture identification and susceptibility testing. The absence of T2 detection in these cases was attributed to the limited pathogen spectrum of the T2Bacteria Panel rather than test failure.

Among the 14 patients, a total of 17 pathogens were isolated (Table 3). Twelve of these belonged to the spectrum of organisms covered by the T2Bacteria Panel, while five were outside its range. The on-panel pathogens comprised *K. pneumoniae* (*n* = 6), *A. baumannii* (*n* = 3), and *P. aeruginosa* (*n* = 3). Pathogens detected only by conventional blood culture and not included in the T2 spectrum were *Burkholderia cepacia* (*n* = 2) and *Staphylococcus epidermidis* (*n* = 3). Three cases of polymicrobial sepsis were observed: one case where *K. pneumoniae* and *P. aeruginosa* were isolated by both methods; one case where *K. pneumoniae* and *A. baumannii* were isolated by the T2Bacteria Panel while blood culture isolated only *K. pneumoniae*; and one case where *K. pneumoniae* and *B. cepacia* were isolated from the blood culture while the T2Bacteria Panel detected only *K. pneumoniae.* In the case where *A. baumannii* was not recovered from blood cultures despite being detected by T2, this likely reflected reduced culture sensitivity in the context of prior antimicrobial exposure, rather than the absence of the organism. The patient demonstrated a severe clinical course consistent with a polymicrobial infection, and antimicrobial therapy was adjusted accordingly.

### 3.3. Diagnostic Performance

The diagnostic performance of the T2Bacteria Panel and blood culture, including ESKAPE pathogens and all species, is shown in the following table (Table 4).

### 3.4. T2Resistance Panel Results

The T2Resistance Panel was applied to all patients with a positive T2Bacteria Panel result. Resistance gene detection was positive in four cases (40%) with *K. pneumoniae* infections, identifying carbapenemase and extended-spectrum β-lactamase (ESBL)-mediated resistance patterns (Table 5).

The results obtained with the T2Resistance Panel were compared with those of phenotypic antimicrobial susceptibility testing from corresponding blood culture isolates (Table 6). In all assessable cases involving *K. pneumoniae*, the molecular resistance markers detected by T2Resistance were fully concordant with the phenotypic resistance profiles. Specifically, the OXA-48 and CTX-M genes were detected in two *K. pneumoniae* isolates, KPC and CTX-M in one isolate, and CTX-M alone in one isolate. In all four cases, the detection of carbapenemase- and ESBL-associated genes corresponded to phenotypic resistance to β-lactam antibiotics.

In contrast, no resistance genes were detected by the T2Resistance Panel in *A. baumannii* and *P. aeruginosa* isolates, despite phenotypic resistance to carbapenems. These discrepancies can be explained by resistance mechanisms that are not covered by the T2Resistance Panel, including OXA-type carbapenemases in *A. baumannii* and predominantly chromosomal, non-enzymatic resistance mechanisms in *P. aeruginosa*.

### 3.5. Antibiotic Susceptibility and Resistance

Analyzing the results of antibiotic susceptibility and resistance testing among isolates of conventional blood cultures, it was observed that 66.7% of *S. epidermidis* strains were methicillin-resistant and 33.3% ciprofloxacin-resistant, while all strains were susceptible (100%) to vancomycin, teicoplanin, amikacin, and linezolid.

Both strains of *B. cepacia* were resistant to trimethoprim/sulfamethoxazole and susceptible to ceftazidime and meropenem.

**Figure 1:**
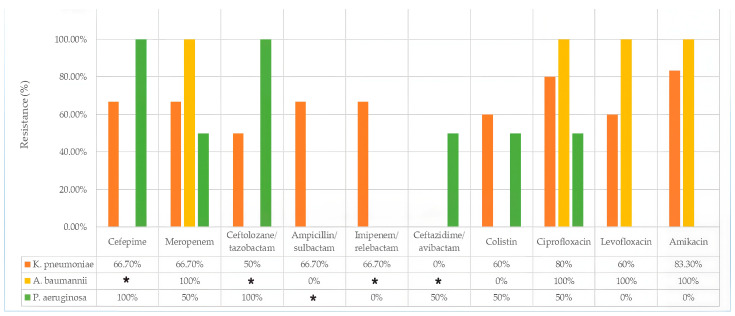
Antibiotic resistance of isolates from conventional blood cultures; * the strain was not tested for susceptibility.

Figure 1 shows the antibiotic resistance of isolated strains of *K. pneumoniae*, *P. aeruginosa*, and *A. baumannii.*

### 3.6. Impact on Time to Results and Therapy

The impact of T2 on patient management was evaluated in terms of the time to results (TTR) and switch from empirical to directed therapy. It was found that, for a T2+/BC+ patients, the median TTR for T2 was 4 h (IQR 3.6–4.8) and that for BC was 108 h (IQR 72.0–168.0). The median time to results (TTR) for the T2Bacteria Panel across all T2-positive cases (*n* = 10) was 4.06 h (IQR 3.5–4.8 h). In comparison, the median time to blood culture positivity and organism identification across all blood culture-positive cases (*n* = 12) was 103 h (IQR 91–120 h).

**Figure 2:**
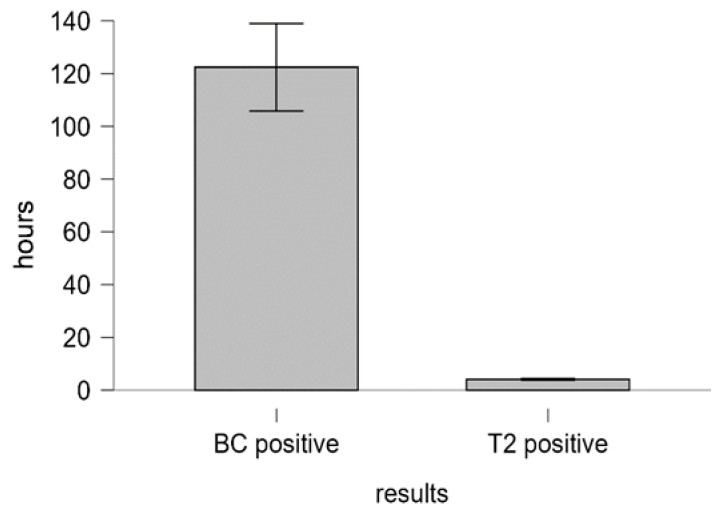
TTR (median, IQR) of positive T2Bacteria Panel (T2) and positive blood culture (BC). Time to results is the interval between the time at which a sample was collected and the time at which T2 or BC results were reported.

Figure 2 shows that the time to results for T2-positive samples was significantly shorter than that for BC-positive cases (*p* < 0.001, Mann–Whitney U test).

Empirical broad-spectrum antibiotic therapy was initiated in all patients at the time of suspected infection. A switch from empirical to directed therapy was observed in 6/10 (60%) T2-positive patients (five T2+/BC+ and one T2+/BC-) and in 5/16 (31.2%) T2-/BC- patients. The switch was observed in 2/4 (50%) patients positive only according to BC. Overall, antimicrobial therapy modifications were closely aligned with diagnostic result availability. In all cases in which treatment was changed based on either T2Bacteria or blood culture findings, adjustments were implemented within a few hours of result reporting, typically within 1–4 h, indicating the rapid clinical integration of microbiological data.

**Figure 3:**
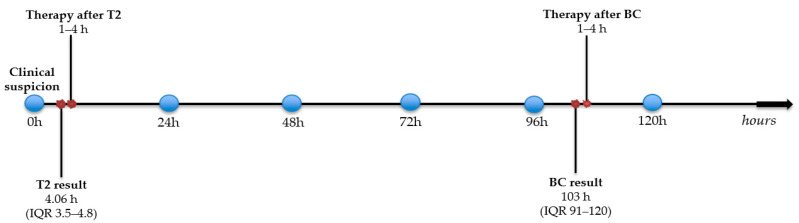
Median diagnostic timeline from clinical suspicion and sampling to microbiological results and antimicrobial therapy modification.

Figure 3 illustrates the median timeline from clinical suspicion and sampling to T2 and blood culture results and subsequent antimicrobial therapy modification.

Table 7 shows the clinical, laboratory, and antimicrobial therapy data of 10 patients with positive T2 results, including resistance gene detection and exact time intervals from sampling to T2 reporting and from result availability to therapy modification, as well as complete blood culture species identification and corresponding time to blood culture positivity.

The types of interventions included targeted escalation to ceftazidime/avibactam in patients with *K. pneumoniae* and detected carbapenemase genes (*n* = 3), de-escalation through the discontinuation of unnecessary Gram-positive coverage (*n* = 2), and the optimization of Gram-negative therapy based on pathogen identification and clinical status (*n* = 1). In the remaining four T2-positive patients, empirical therapy was not modified because the initial treatment already provided adequate pathogen coverage (*n* = 2), broad-spectrum therapy was maintained due to clinical severity and hemodynamic instability (*n* = 1), or treatment adjustments were performed later based on blood culture susceptibility results rather than the initial T2 findings (*n* = 1).

### 3.7. Clinical Outcomes and 30-Day Mortality

The overall 30-day mortality rate in the study cohort was 13/30 (43.3%). Mortality was analyzed descriptively across diagnostic and therapeutic subgroups due to the limited sample size. According to the test result concordance, 30-day mortality occurred in two patients with concordant positive results (T2+/BC+), in one patient with T2-negative/blood culture-positive findings (T2−/BC+), and in 10 patients with concordantly negative results (T2−/BC−). No deaths were observed among patients with T2-positive/blood culture-negative results. Among patients who died, antimicrobial therapy had been modified following T2 results in two cases and after blood culture reporting in an additional five cases, whereas no treatment changes were performed in the remaining patients. Deaths associated with microbiologically confirmed bloodstream infections were related to *K. pneumoniae* harboring carbapenemase or ESBL-associated resistance genes (KPC, OXA-48, CTX-M) (*n* = 2) and one case of *S. epidermidis* bacteremia. Due to the small number of events within individual subgroups, no formal statistical comparisons were performed, and these findings should be interpreted as descriptive and exploratory.

## 4. Discussion

Our results are in line with prior prospective multicenter studies evaluating the T2Bacteria Panel. In a pivotal diagnostic accuracy study of 1427 patients across 11 U.S. hospitals, Nguyen et al. reported sensitivity of 90%, specificity of 98%, and an NPV of 99.7%, with a mean time to result that was 5.4 h–66 h faster than for conventional blood cultures [17]. Similarly, Drevinek et al. demonstrated the excellent sensitivity and specificity of the T2Bacteria Panel in detecting ESKAPE pathogens directly from whole blood, further validating its clinical performance [21]. Our data, while from a smaller single-center cohort, echo these findings. The T2Bacteria Panel demonstrated sensitivity of 83%, specificity of 100%, and a negative predictive value of 94%, with a median time to result of 4.06 h, compared with 103 h for conventional blood cultures.

The specific characteristics of the cardiovascular surgical population should be considered when interpreting these findings. Following cardiac surgery, patients frequently exhibit a pronounced sterile inflammatory response related to cardiopulmonary bypass and surgical trauma, which may mimic sepsis and increase diagnostic uncertainty [18]. In addition, empirical broad-spectrum antimicrobial therapy is commonly initiated early in this population, potentially reducing blood cultures’ sensitivity [16]. These factors may partly explain the preserved diagnostic yield of the T2Bacteria Panel and the high proportion of culture-negative cases observed in our cohort compared with general ICU populations.

Discordant results require careful clinical interpretation. In T2-positive/blood culture-negative cases, all patients had received prior antimicrobial therapy, and the clinical improvement after targeted treatment supported the likelihood of a true bloodstream infection rather than false-positive molecular detection. Conversely, all T2-negative/blood culture-positive cases were caused by organisms outside the T2Bacteria Panel’s spectrum, emphasizing that negative T2 results do not exclude infection with off-panel pathogens and should always be interpreted in conjunction with clinical assessment and conventional culture results [10].

The detection of resistance genes by T2Resistance also aligns with the emerging literature. Ashcraft et al. recently demonstrated the utility of the T2Resistance Panel in identifying clinically relevant resistance determinants within hours, compared to 2–4 days required for phenotypic susceptibility testing [12]. Our study adds to this growing body of evidence by showing that T2Resistance identified OXA-48, KPC, and CTX-M genes in patients with *K. pneumoniae*—results that were consistent with phenotypic resistance patterns.

In our cohort, the molecular resistance markers detected by the T2Resistance Panel were fully concordant with the phenotypic susceptibility results for *K. pneumoniae* isolates, supporting the reliability of early resistance gene detection for clinical decision-making [12].

Beyond diagnostic accuracy, the clinical impact of T2 assays has been demonstrated in stewardship-focused studies. Bookstaver et al. showed that combining rapid diagnostics with antimicrobial stewardship interventions reduced the time to effective therapy and enabled earlier de-escalation [10]. Consistent with these findings, our study demonstrated that therapeutic modifications were made in nearly one quarter of patients based on the T2 results, highlighting the real-world impact of this technology in optimizing antimicrobial therapy.

The rapid turnaround time of the T2Bacteria and T2Resistance Panels is of particular clinical importance. In patients with sepsis and septic shock, every hour of delay in appropriate therapy is associated with a 7.6% decrease in survival [3]. Conventional blood cultures, with a median time to species identification exceeding 40 h [15], are often too slow to inform initial therapeutic decisions, necessitating empiric broad-spectrum coverage. By providing results within 4–7 h, the T2Bacteria Panel bridges this critical gap, allowing for earlier pathogen-directed therapy.

In our study, the median time advantage of more than 100 h compared with conventional blood cultures translated into earlier therapeutic optimization. Such a time gain is clinically meaningful, as it allows rapid escalation in cases of multidrug-resistant pathogens or early de-escalation when broad-spectrum coverage is unnecessary, potentially reducing toxicity, antimicrobial selection pressure, and healthcare costs [9,10].

In our cohort, therapeutic changes were made in six of ten T2-positive patients (60%) based on the T2 results. In the remaining patients, empirical therapy was not modified because the initial treatment already provided adequate pathogen coverage, clinical instability required the continuation of broad-spectrum therapy, or treatment decisions were deferred until full susceptibility results became available. These included both escalation to cover multidrug-resistant pathogens and de-escalation when unnecessary coverage could be safely discontinued. Such interventions not only improve individual patient outcomes but also contribute to broader public health goals by reducing selective pressure for antimicrobial resistance.

The high negative predictive value observed in our study also has important stewardship implications. Concordantly negative T2 and blood culture results supported early diagnostic reassessment, the discontinuation of unnecessary anti-ESKAPE coverage, and the redirection of the diagnostic workup toward non-bacterial causes of systemic inflammation or alternative infection sites [9,10].

From a practical perspective, the clinical interpretation of the combined results may be summarized as follows: concordant positive results (T2+/BC+) support early targeted therapy; T2-positive/blood culture-negative findings should prompt early treatment adjustment when supported by clinical signs; T2-negative/blood culture-positive cases require therapy guided by culture results, particularly for off-panel organisms; and concordantly negative results (T2−/BC−) should encourage the consideration of de-escalation and the reassessment of the infection diagnosis.

Despite its promising results, this study has several important limitations. First, the small sample size (*n* = 30) limited the statistical power of the analysis and precludes definitive conclusions regarding clinical outcomes such as mortality, length of stay, or cost-effectiveness. Larger multicenter trials are needed to validate these findings. Second, the study was retrospective and conducted at a single cardiovascular ICU, which may limit inference. The prevalence of on-panel pathogens and the antimicrobial resistance patterns observed in our cohort may not reflect other clinical settings.

Another limitation lies in the predefined spectrum of the T2Bacteria Panel. While it demonstrated high sensitivity for the target pathogens, it failed to detect two clinically significant organisms (*B. cepacia* and *S. epidermidis*), which were isolated exclusively by blood cultures. This highlights the complementary role of blood culture, which remains indispensable in detecting off-panel organisms and in performing comprehensive antimicrobial susceptibility testing. Finally, cost considerations were not assessed in this study, although prior economic analyses have suggested that rapid diagnostics may be cost-neutral or even cost-saving when accounting for reductions in length of stay and inappropriate antimicrobial use [22].

The integration of rapid molecular diagnostics into routine sepsis management represents a paradigm shift. Future directions include expanding the spectrum of the T2Bacteria Panel to incorporate a broader range of pathogens, including coagulase-negative staphylococci and less common Gram-negative organisms. Additionally, combining T2 results with those of other rapid diagnostic technologies, such as multiplex PCR panels, metagenomic sequencing, or next-generation phenotypic assays, may provide even more comprehensive diagnostic coverage.

From a clinical perspective, prospective multicenter studies are needed to evaluate the impact of T2-guided therapy on hard outcomes, such as mortality, the length of the ICU stay, antimicrobial utilization, and healthcare costs. The integration of T2 testing into structured antimicrobial stewardship programs will be critical in maximizing its benefits. Furthermore, cost-effectiveness analyses across diverse healthcare systems will help to determine the broader feasibility of widespread implementation.

## 5. Conclusions

This single-center retrospective study demonstrates that the T2Bacteria Panel enables the rapid, highly accurate detection of bloodstream pathogens within its spectrum, substantially reducing the time to microbiological diagnosis compared with conventional blood cultures. Combined with the T2Resistance Panel, this approach supported the earlier optimization of antimicrobial therapy and facilitated antimicrobial stewardship, reducing unnecessary broad-spectrum use.

Nevertheless, the limitations of this study—including the small sample size, the single-center design, and the restricted pathogen spectrum of the T2Bacteria Panel—must be acknowledged. Conventional blood cultures remain indispensable in detecting off-panel pathogens and for comprehensive susceptibility testing.

Negative T2 results should be interpreted with caution, as bloodstream infections caused by organisms outside the panel’s spectrum may occur. Therefore, empirical antimicrobial therapy should not be modified solely on the basis of a negative T2 result, and conventional microbiological testing together with clinical assessment remains essential for treatment guidance.

Future studies should focus on clinically meaningful outcomes, including the impact of T2-guided management on antimicrobial stewardship and de-escalation and patient-centered outcomes such as mortality and length of stay, as well as cost-effectiveness in high-risk populations.

## Figures and Tables

**Table 1:** Baseline demographic and clinical characteristics of the study cohort (*n* = 30).

**Patients**	
Years, Median (IQR)	65.5 (50–70)
Gender, *n* (%)	Male: 19 (63.3%), Female: 11 (36.7%)
Type of admission, *n* (%)	Emergency: 6 (20%), Elective: 24 (80%)
Type of surgery, *n* (%)	Cardiac: 29 (96.7%), Vascular: 1 (3.3%)
**Laboratory Parameters**	
Leukocytes, cells ×10^9^/L, Median (IQR)	12.45 (9.72–15.2)
Leukocytosis, >12 ×10^9^/L, *n* (%)	15 (50.0%)
Leukopenia, <4 ×10^9^/L, *n* (%)	3 (10.0%)
CRP, mg/dL, Median (IQR)	142.0 (107.4–257.9)
PCT, ng/mL, Median (IQR)	2.34 (0.89–11.13)
Lactate, mmol/L, Median (IQR)	1.55 (1.2–2.15)
**Clinical Data**	
Body temperature, °C, Median (IQR)	38.1 (36.93–38.8)
Temperature > 38 °C, *n* (%)	18 (60.0%)
Temperature < 36 °C, *n* (%)	3 (10.0%)
Heart rate, bpm, Median (IQR)	104.5 (88.2–115)
Mean arterial pressure, mmHg, Median (IQR)	76 (71–86)
Glasgow Coma Scale, Median (IQR)	14 (11–15)
Sequential Organ Failure Assessment Score, Median (IQR)	8.5 (6–11)
30-day mortality, *n* (%)	13 (43.3%)
**Concomitant Diseases**	
Hypertension, *n* (%)	28 (93.3%)
Chronic renal failure, *n* (%)	11 (36.6%)
Diabetes mellitus, *n* (%)	10 (33.3%)
Chronic lung disease, *n* (%)	2 (6.6%)
Smoking, *n* (%)	12 (40.0%)

Data refer to the time of T2 and BC sample collection, with the exception of mortality. CRP, C-reactive protein; IQR, interquartile range; PCT, procalcitonin.

**Table 2:** Comparison of T2Bacteria Panel and blood culture results.

	BC Positive	BC Negative	Total
T2 Positive	8	2	10
T2 Negative	4	16	20
Total	12	18	30

**Table 3:** Distribution of pathogens detected by T2 and blood culture (total isolates, *n* = 17).

Pathogen	Number of Isolates
*K. pneumoniae*	6
*A. baumannii*	3
*P. aeruginosa*	3
*B. cepacia*	2
*S. epidermidis*	3

**Table 4:** Diagnostic performance of T2 and blood culture.

Method	Sensitivity (95% CI)	Specificity (95% CI)	PPV (95% CI)	NPV (95% CI)
T2Bacteria (within-panel)	100.0% (73.5–100.0)	100.0% (78.2–100.0)	100.0%	100.0%
T2Bacteria (all species)	66.7% (34.9–90.1)	88.9% (65.3–98.6)	80.0% (44.4–97.5)	80.0% (56.3–94.3)
Blood Culture	75.0% (42.8–94.5)	100.0% (78.2–100.0)	100.0%	84.2% (60.4–96.6)

PPV—positive predictive value; NPV—negative predictive value; CI—confidence interval.

**Table 5:** T2Resistance Panel results.

Patient Group (*n* = 10)	Resistance Gene(s) Detected	Frequency
OXA-48 + CTX-M	2 patients	20%
KPC + CTX-M	1 patient	10%
CTX-M only	1 patient	10%
No resistance genes	6 patients	60%

KPC—Klebsiella pneumoniae carbapenemase; OXA-48—oxacillinase-48 carbapenemase; CTX-M—cefotaximase-M extended-spectrum β-lactamase (ESBL).

**Table 6:** Concordance between T2Resistance gene detection and phenotypic antimicrobial susceptibility testing.

Pathogen	T2Resistance Gene Detected	Blood Culture Isolate Available	Phenotypic Susceptibility Result (Blood Culture)	Concordance
*K. pneumoniae*	*blaOXA-48 + blaCTX-M*	Yes	Carbapenem-resistant, ESBL-positive	Yes
*K. pneumoniae*	*bla*OXA-48 + *bla*CTX-M	Yes	Carbapenem-resistant, ESBL-positive	Yes
*K. pneumoniae*	*bla*KPC + *bla*CTX-M	Yes	Carbapenem-resistant, ESBL-positive	Yes
*K. pneumoniae*	*bla*CTX-M	Yes	ESBL-positive	Yes
*A. baumannii*	None detected	Yes	Carbapenem-resistant	Not assessable (off-panel mechanism)
*P. aeruginosa*	None detected	Yes	Multidrug-resistant/carbapenem-resistant	Not assessable (chromosomal mechanisms)

KPC—Klebsiella pneumoniae carbapenemase; OXA-48—oxacillinase-48 carbapenemase; CTX-M—cefotaximase-M extended-spectrum β-lactamase (ESBL).

**Table 7:** Clinical and laboratory details in patients with positive T2Bacteria Panel.

Age, Gender	SOFA Score	T2Bacteria Result	T2Resistance Result	TTR T2 (h)	Time to Modification of Therapy After T2 (h)	Blood Culture Result	TTR BC (h)	Empirical Therapy	Switch to Directed Therapy After T2	Switch to Directed Therapy After Blood Culture/Panel
50, M	8	*K. pneumoniae*	OXA-48 + CTX-M	4	2	*K. pneumoniae*	168	Vancomycin, meropenem	Ceftazidime/avibactam	Same
30, F	2	*K. pneumoniae*	OXA-48 + CTX-M	5	3	*K. pneumoniae*	192	Linezolid, meropenem	Ceftazidime/avibactam	Same
77, M	11	*A. baumannii*	None detected	3	2	*A. baumannii*	120	Vancomycin, meropenem	Meropenem, ampicillin/sulbactam	Same
49, M	13	*K. pneumoniae*	KPC + CTX-M	4	3	*K. pneumoniae*	96	Vancomycin	Ceftazidime/avibactam	Same
62, M	8	*P. aeruginosa*	None detected	3	2	No growth	105	Cefuroxime, meropenem	Meropenem	Same
75, F	6	*K. pneumoniae* *A. baumannii*	None detected	3.5	1	Only *K. pneumoniae*	168	Vancomycin, meropenem	Meropenem	Same
63, F	3	*K. pneumoniae* *P. aeruginosa*	CTX-M	4	2	*K. pneumoniae P. aeruginosa*	192	Cefuroxime, meropenem	Meropenem, amikacin	Ceftazidime/avibactam, vancomycin
65, F	13	*P. aeruginosa*	None detected	4	No change	*P. aeruginosa*	72	Vancomycin, meropenem	No	Piperacillin/tazobactam
79, M	16	*A. baumannii*	None detected	4.5	No change	No growth	92	Linezolid, meropenem	No	Fosfomycin, colistin
47, M	5	*K. pneumoniae*	None detected	5	No change	*K. pneumoniae*	96	Vancomycin, meropenem	No	Ceftazidime

M—male; F—female; KPC—Klebsiella pneumoniae carbapenemase; OXA-48—oxacillinase-48 carbapenemase; CTX-M—cefotaximase-M extended-spectrum β-lactamase (ESBL).

## Data Availability

Data are unavailable due to privacy and ethical restrictions.
